# Effects of comorbid chronic kidney disease on late-onset hypophosphatasia mice under treatment with asfotase alfa

**DOI:** 10.1093/jbmrpl/ziag074

**Published:** 2026-04-17

**Authors:** Cintia Tokuhara, Flavia Amadeu de Oliveira, Sonoko Narisawa, Elis J Lira dos Santos, Brian L Foster, Jose Luis Millan

**Affiliations:** Sanford Children's Health Research Center, Sanford Burnham Prebys Medical Discovery Institute, La Jolla, CA 92037, United States; Sanford Children's Health Research Center, Sanford Burnham Prebys Medical Discovery Institute, La Jolla, CA 92037, United States; Sanford Children's Health Research Center, Sanford Burnham Prebys Medical Discovery Institute, La Jolla, CA 92037, United States; Division of Biosciences, College of Dentistry, The Ohio State University, Columbus, OH 43210, United States; Division of Periodontology, College of Dentistry, The Ohio State University, Columbus, OH 43210, United States; Division of Biosciences, College of Dentistry, The Ohio State University, Columbus, OH 43210, United States; Sanford Children's Health Research Center, Sanford Burnham Prebys Medical Discovery Institute, La Jolla, CA 92037, United States

**Keywords:** preclinical model, mouse, loss-of-function, hypophosphatasia, CKD-mineral and bone disorder

## Abstract

Hypophosphatasia (HPP) is a rare bone disorder caused by loss-of-function mutations in the *ALPL* gene, leading to deficient tissue-nonspecific alkaline phosphatase (TNAP) activity and impaired skeletal/dental mineralization. Asfotase alfa (AA), the only FDA-approved therapy, improves survival and skeletal/dental mineralization. Because of its mineral-binding properties, AA also binds to sites of ectopic calcification, including vasculature. Overexpression of TNAP in either the vascular media or intima has been shown to induce severe calcification. Here, we examined what might occur if a disease that features medial artery calcification, such as CKD-mineral and bone disorder (CKD-MBD), were to develop in a subject with HPP undergoing AA treatment. Two-month-old *Alpl^Prx1/−^* (HPP) or *Alpl^flox/−^* (non-HPP control) mice were treated with s.c. AA injections (8.2 mg/kg, 3×/week) for 4 mo. After 3 mo of treatment, CKD was induced via a 0.2% adenine and 1.8% phosphorus diet. Mice were euthanized at 6 mo of age, and skeletal, renal, cardiac, and vascular tissues were analyzed. Alkaline phosphatase levels slightly increased with AA but significantly increased after inducing CKD in control and HPP female mice. In males, ALP activity was elevated in the AA and AA+CKD cohorts. Micro-CT analysis showed improved bone parameters in HPP under AA treatment. However, the improvements were dampened when CKD was induced, compared to non-CKD *Alpl^flox/−^*. Immunohistochemistry of the kidney revealed increased TNAP immunolocalization in the AA+CKD cohort, in both females and males. Ectopic renal and vascular calcification was observed in the AA+CKD group. Gene expression profiling in the kidney revealed that AA treatment modulates the immune and mineral metabolism pathways, while CKD superimposition induces a shift toward pro-inflammatory and pro-fibrotic responses, compromising renal and skeletal homeostasis. These findings underscore the importance of carefully evaluating HPP patients under treatment with mineral-targeted TNAP at risk of developing comorbid conditions associated with ectopic calcification.

## Graphical Abstract

**Graphical Abstract:**
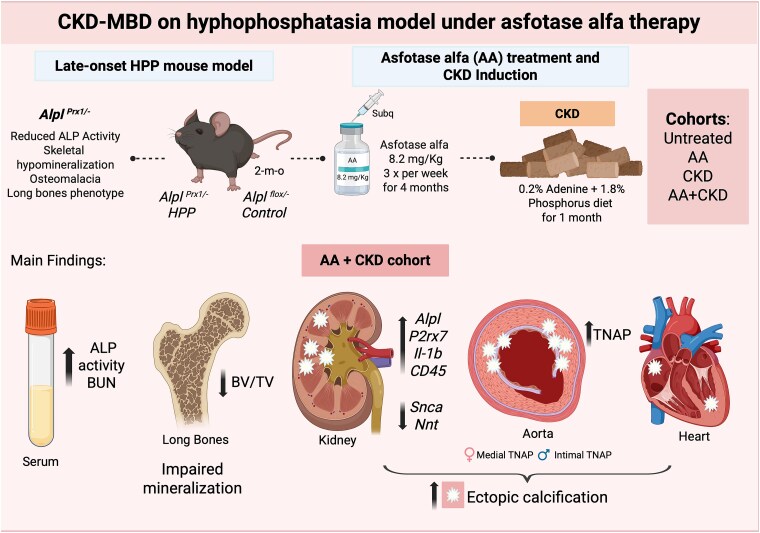

## Introduction

Hypophosphatasia (HPP) is a rare, inherited metabolic bone disorder caused by loss-of-function mutations in the *ALPL* gene, which encodes tissue-nonspecific alkaline phosphatase (TNAP).^1^ Tissue-nonspecific alkaline phosphatase plays a key role in bone and dental mineralization by hydrolyzing inorganic pyrophosphate (PP_i_), a potent endogenous inhibitor of hydroxyapatite crystal formation.^2^ Deficiency of TNAP activity results in PP_i_ accumulation, leading to impaired skeletal and dental mineralization, increased fracture risk, premature tooth loss, and, in severe cases, respiratory failure and early death.^1^

Asfotase alfa (AA) is a mineral-targeted recombinant human TNAP that has demonstrated efficacy in improving survival and promoting skeletal/dental mineralization in HPP in perinatal and pediatric-onset forms.^3^ To date, enzyme replacement therapy (ERT) for HPP is approved only for adults with pediatric-onset disease, except in Japan, where ERT is permitted regardless of the age at disease onset.^4^ Adult HPP patients often exhibit persistent and debilitating symptoms, including chronic pain, muscle weakness, stress fractures, and compromised mobility, which can significantly impair quality of life.^5^

Importantly, as HPP patients age, they may be more likely to develop comorbidities common to the general aging population, such as CKD,^6^ and degenerative joint diseases.^7^ The potential interactions between AA therapy and age-associated disorders, particularly those involving mineral metabolism and inflammation, remain poorly understood. Because AA binds to sites of active mineral deposition, including ectopic calcification in soft tissues and the vasculature,^8^ there is concern that the presence of comorbid conditions, such as CKD-mineral and bone disorder (CKD-MBD), may have an impact on treatment effectiveness.^9^ CKD-MBD is a leading contributor to cardiovascular mortality in dialysis patients, characterized by bone disorders, ectopic calcification, and various biochemical imbalances. The decline in kidney function disrupts hormonal regulation, including elevated levels of PTH and fibroblast growth factor 23 (FGF-23), along with reduced calcitriol. These endocrine disturbances contribute to abnormal bone turnover and the development of ectopic calcification.^10^ Experimental evidence shows that overexpression of TNAP in vascular tissues can induce severe medial arterial calcification,^11^ yet the effect of systemic TNAP replacement via AA in patients with existing vascular pathology is unknown. This is particularly relevant given the adult HPP population and the increasing use of long-term AA treatment in this group.

In this study, we aimed to investigate the consequences of superimposing CKD on a murine model of adult-onset HPP undergoing long-term treatment with AA. Using *Alpl^Prx1/−^* (HPP) mice, we examined skeletal outcomes, renal and vascular calcification, following AA treatment alone or in combination with CKD induction. Our findings reveal that the presence of CKD as a comorbidity affects the efficacy of the ongoing treatment.

## Materials and methods

### Animal care

The study received approval from the Sanford Burnham Prebys Medical Discovery IACUC (AUF #22-048). The animals were bred and maintained according to the animal facility’s standard operating procedures for breeding. Mice were housed in a clean, pathogen-free environment with a 12-h light/dark cycle. A maximum of 5 animals were housed per cage and had free access to a regular and special diet. Exsanguination with anesthesia or CO_2_ was performed following IACUC regulations and the American Veterinary Medical Association (AVMA) Guidelines on Euthanasia.

### Treatment regimens and cohorts

Eight-week-old *Alpl^flox/−^* and *Alpl^Prx1/−^* mice on a C57BL/6 background were used as non-HPP controls and late-onset HPP mice, respectively. The *Alpl^Prx1/−^* mice were generated via conditional ablation of one allele of the *Alpl* gene and the second *Alpl* allele constitutively deleted in the mesenchymal cells through the expression of Cre recombinase under the control of the *Prx1* promoter.^12^ Asfotase alfa was injected s.c. three times per week at a concentration of 8.2 mg/kg of body weight (BW) for 4 mo. To induce CKD, mice were given a 0.2% adenine diet (Teklad Custom Diet TD.09138, Envigo) for 3 wk which was then supplemented with a high (1.8%) phosphorus diet (Teklad Custom Diet TD.190831, Envigo) for an additional week to induce kidney damage, uremia, and hyperphosphatemia, adapted from Mohamed et al.^13^ Mice were euthanized at 6 mo of age, and skeletal and soft tissues were collected for the following experiments.

### Native gel electrophoresis for alkaline phosphatase activity

Kidneys from HPP and non-HPP mice in the presence or absence of CKD were collected as well as liver, bone, and kidney tissues from a WT mouse. Samples were homogenized and extracted using the butanol extraction method, and total protein concentration was determined by bicinchoninic acid assay. For comparison of migration patterns of alkaline phosphatase (ALP), 20 μg of total protein from kidney samples or adjusted amounts from liver, bone, and kidney from the WT (normalized for ALP activity) were loaded per lane. Native polyacrylamide gel electrophoresis (PAGE) was performed without denaturing agents to preserve enzymatic activity. Following electrophoresis, ALP activity was detected using the Azo Dye coupling method. A molecular weight marker was run in parallel, with the 250 kDa protein band indicated in blue.^14^

### Biochemical analysis

Serum and plasma were collected from *Alpl*^flox/−^ and *Alpl*^Prx1/−^ mice using Pasteur pipettes through the orbital sinus up to 180 d postnatal (dpn). Afterward, the blood samples were centrifuged at 5200 × *g* for 10 min. For the PP_i_ assay, deproteinization was conducted by adding 20 μL of plasma to a Microcon-10 kDa Centrifugal Filter Unit with Ultracel-10 membrane (MilliporeSigma, Merck KGaA), followed by centrifugation at 14 000 × *g* for 20 min. Inorganic pyrophosphate concentration was measured as previously reported.^15^^,^^16^ The ALP activity assay was performed by adding 5 μL of serum and recombinant human ALP standards (216.0-0.0099 ng/mL) into a 96-well plate and followed by the addition of 95 μL of 10 mM pNPP in diethanolamine (DEA) buffer (pH 9.8) containing 1.0 mM MgCl_2_ and 20 μM ZnCl_2_ Also, the absorbance of the kinetics assay was measured at 405 nm using OptiMax Microplate Absorbance Reader (Molecular Devices, LLC), for 30 min.^16^

### Blood urea nitrogen and liver profile

To confirm renal damage and establish the CKD condition, 100 μL of fresh whole blood were used to measure blood urea nitrogen (BUN; reference range: 8-33 mg/dL) and liver biochemical parameters, including alanine aminotransferase (ALT; 17-77 U/L), gamma-glutamyl transferase (GGT; 6-9 U/L), bile acids (BA; μmol/L), total bilirubin (TBIL; 0.2-0.5 mg/dL), and total cholesterol (CHOL; 81-135 mg/dL), using Abaxis VetScan Mammalian Liver Profile rotors on a VetScan VS2 chemistry analyzer (Zoetis).

### Tissue collection and processing for histological assay

Mice were anesthetized with isoflurane and euthanized by exsanguination at 6 mo of age. Hard and soft tissues were collected, fixed in 4% paraformaldehyde/PBS solution, and processed for histological analysis. The long bones were placed in 0.125 M EDTA/10% formalin (pH 7.3) solution for 7 d for decalcification, then paraffin-embedded and sectioned at 5 μm.

### Radiography and micro-CT analysis

The Faxitron MX-20DC4 radiography system (Chicago, IL, USA) provided the radiographic images of the skeletal tissue with an energy of 20 kV. The femur and tibia lengths were measured by ImageJ (Rasband WS, ImageJ, National Institutes of Health).

Micro-CT (μCT) was performed on 4% paraformaldehyde/PBS solution-fixed samples, which were scanned in a μCT (Scanco Medical) at 70 kV, 114 μA, 0.5 mm Al filter, 900 ms integration time, and 10 μm voxel size. Femurs were analyzed according to standard trabecular and cortical bone analysis using a bone threshold of 400 and 550 mg HA/cm^3^, respectively.^13^ A set of 5 hydroxyapatite phantoms of known density was used to calibrate the scans. Reconstructed DICOM files were exported and analyzed using Analyze version 14.0 (AnalyzeDirect).^17^

### Von Kossa staining, immunohistochemistry, and immunofluorescence assay

The Von Kossa staining was performed according to standard methods,^18^ and the slides were scanned using the Aperio AT2 system (Leica Biosystems of Leica Microsystems Inc.).^19^ The quantification of the ectopic calcification was determined by Aperio ImageScope version 12.4.6. The ectopic calcification spots were measured using the Positive Pixel Count v9 algorithm, considering only the number of strong positive pixels, and then converted to percentages using the following equation: (number of strong positive pixels × 100%)/sum of total pixels (including weak, positive, strong, and negative pixels).

Immunohistochemistry (IHC) was performed as previously described.^20^ The rat monoclonal anti-TNAP (#MAB2909, R&D) was used as a primary antibody. The antibody’s localization was detected using the ABC method (avidin-biotin complex), which amplifies the signal by binding a biotinylated secondary antibody to an avidin-peroxidase complex (Vector Laboratories). Goat anti-rat and secondary antibodies were used. Hematoxylin was used as a counterstain. Quantitative data were measured using ImageJ.

Immunofluorescent staining of formalin-fixed paraffin-embedded tissue (FFPE-IF) was performed as follows. After deparaffinization, rehydration, a polyclonal goat anti-ALPL (#AF2910, R&D) was used as the primary antibody, followed by the secondary Alexa Fluor 488 donkey anti-goat IgG (H+L) (A11055, Invitrogen). The images were acquired by the Zeiss LSM 710 confocal microscope system.

### RT-qPCR

Real-time qPCR was performed to investigate the gene expression of CKD markers in the late-onset HPP mouse model. Thirty milligrams of whole kidney tissue were homogenized in 700 μL of RNA lysis buffer (RLT) along with 1% β-mercaptoethanol. The lysate was centrifuged for 3 min at full speed, followed by the collection of the supernatant. The purification of total RNA was performed according to the RNeasy Mini Kit (Qiagen, Valencia, #74104), and after RNA quantification by Nanodrop (Nanodrop Technologies), 2 μg of RNA was reverse transcribed, with 0.1 μg cDNA was used for qPCR reactions. The reverse transcriptase RT-qPCR was performed using SensiMix Hi-Rox SyBR Green, and the results were normalized to *Actb* as the housekeeping gene.^16^ List of used primers can be found in the Table S1.

### Statistical analysis

The statistical analysis was performed using GraphPad Prism version 10.2.0 (GraphPad Software), with *n* = 3-13 mice per group. The data were presented as mean ± SD, and the statistical analysis was carried out using an unpaired *t*-test for comparisons between 2 groups or one-way ANOVA followed by Tukey’s multiple comparison test for more than 2 groups. Significance was established at *p* < .05.

## Results

### Biochemical markers and hepatic response to AA in the HPP and CKD superimposed mice

**Figure 1:**
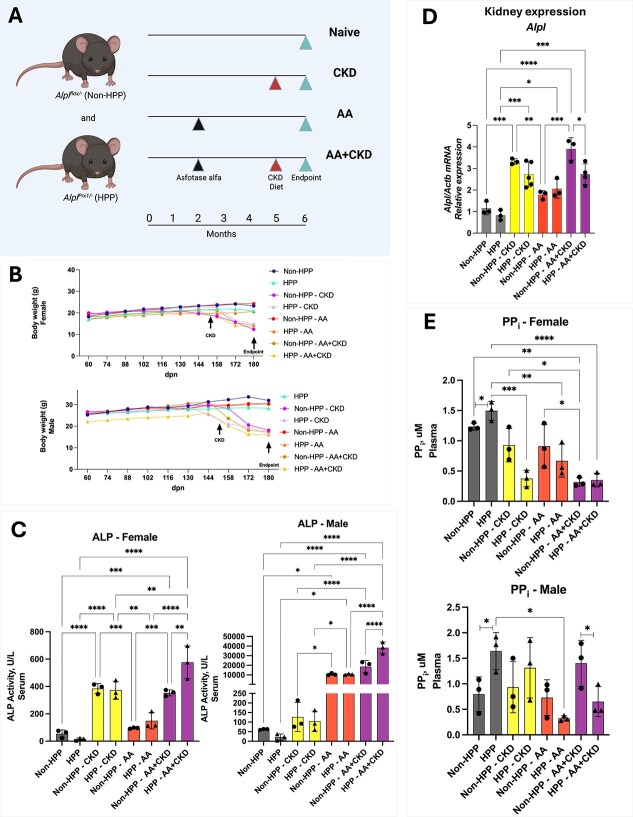
Biochemical and renal *Alpl* gene expression responses to asfotase alfa (AA) in a late-onset HPP mouse model with superimposed CKD. (A) Experimental design. *Alpl^Prx1/−^* (HPP) and *Alpl^flox/−^* (non-HPP) mice were treated with AA (8.2 mg/kg, s.c., three times per week) starting at 2 mo of age. At 5 mo, CKD was induced via a 0.2% adenine-enriched diet for 3 wk, followed by a 0.2% adenine plus 1.8% phosphate-enriched diet for an additional week. Mice were euthanized at 6 mo of age for analysis. (B) Body weight was monitored over time. (C) Serum alkaline phosphatase (ALP) activity. (D) *Alpl* gene expression in the kidney of male mice. (E) Inorganic pyrophosphate (PP_i_) levels. *N* = 3. Data are presented as mean ± SD. One-way ANOVA with Tukey’s post hoc test was used. *p* < .05 (^*^), *p* < .01 (^**^), *p* < .001 (^***^), *p* < .0001 (^****^).

The following 4 cohorts, and a total of 8 groups of mice were analyzed. *Control cohort*: *Alpl^Prx1/−^* (also called here, HPP mice) and *Alpl^flox/−^* (also called here, non-HPP) mice fed a regular diet for the entire 6-mo period); *CKD cohort*: *Alpl^Prx1/−^* and *Alpl^flox/−^* mice fed regular diet for 5 mo followed by a 3 wk feeding on a 0.2% adenine followed by a 1 wk feeding with a 0.2% adenine plus 1.8% phosphorus; *AA cohort*: *Alpl^Prx1/−^* and *Alpl^flox/−^* mice fed a regular diet for the entire 6 mo period and injections of AA 3×/wk starting at 2 mo of age until euthanasia; *AA+CKD cohort*: *Alpl^Prx1/−^* and *Alpl^flox/−^* mice fed a regular diet for 5 mo followed by a 3 wk feeding on a 0.2% adenine followed by a 1 wk feeding with a 0.2% adenine plus 1.8% phosphorus, with injections of AA 3×/wk starting at 2 mo of age until euthanasia (Figure 1A). Body weight was monitored continuously throughout the experiment. Both females and males had a similar profile with sustained BW until 150 d, when it began to decrease in the CKD only and AA+CKD (Figure 1B).

A biochemistry panel showed that ALP levels were significantly higher after induction of CKD; however, no differences were observed after AA treatment in female non-HPP and HPP mice. In addition, ALP levels in HPP mice were higher than in non-HPP control mice when treated with AA plus the CKD diet (Figure 1C). In males, ALP levels differed somewhat from those in females, with higher levels in the AA group and the AA+CKD cohort compared with untreated mice. ALP levels in HPP mice were higher than in non-HPP mice when treated with AA plus CKD at 180 dpn (Figure 1C). Given that the same dose of AA was administered to both female and male non-HPP and HPP mice, the high ALP levels in the CKD and AA+CKD cohorts suggested an endogenous upregulation of *Alpl* expression. As TNAP, besides bone, is known to be highly expressed in the kidney, we surmised that high ALP activity in blood could be derived from upregulation of kidney *Alpl* as a result of inducing CKD. As shown in Figure 1D, there was upregulation of male murine *Alpl* gene expression in the kidney in non-HPP CKD and HPP+CKD male mice, and also in AA+CKD mice in both non-HPP and HPP groups, and native PAGE showed that the enzyme present in kidney had the characteristic migration pattern of the normal kidney TNAP isoform (Figure S1). In females and males, PP_i_ levels were higher in control HPP mice when compared to the non-HPP control mice. In female HPP mice, PP_i_ levels were lower in the CKD, AA, and AA+CKD cohorts than in control HPP mice. In males, PP_i_ levels were reduced in HPP mice treated with AA compared with HPP mice. In the AA+CKD HPP cohort, PP_i_ levels were lower than AA+CKD non-HPP mice at 180 dpn (Figure 1E). Phosphorus showed high levels after treatment with the high-phosphorus diet in both CKD and AA+CKD cohorts compared to untreated controls. Results from female HPP mice showed higher phosphorus levels in the CKD group when compared to non-HPP mice. No changes were observed in either female or male AA-treated mice compared to Non-HPP and HPP controls (Figure S2A). Regarding the males, the phosphorous levels were higher in Non-HPP-AA+CKD group compared to Non-HPP control (Figure S2A). In addition, we did not find significant differences in serum calcium levels between HPP and non-HPP mice across groups (Figure S2B).

Liver biochemical profiles and BUN levels were assessed in both non-HPP and HPP mice exposed to AA, CKD, or their combination (AA+CKD) (Table S2). Blood urea nitrogen levels were markedly elevated, above the reference range (8-33 mg/dL) in all CKD and AA+CKD groups, confirming renal dysfunction. The AA-treated group was in the normal range (Table S2). In HPP mice, female CKD (148 mg/dL) and AA+CKD (107.3 mg/dL), as well as male CKD (86 mg/dL) and AA+CKD (100 mg/dL), exhibited consistently high BUN values. ALT, a marker of hepatocellular injury, was significantly increased in non-HPP mice with AA+CKD (female: 980 U/L; male: 937 U/L), whereas HPP mice showed higher levels compared to the normal range values of 17-77 U/L, but attenuated responses (AA+CKD: female: 93.5 U/L; male: 255 U/L) compared to non-HPP, suggesting a differential hepatic response in the context of TNAP deficiency. Gamma-glutamyl transferase levels in females were higher in the non-HPP-AA+CKD group (69 U/L). Bilic acid was elevated in the CKD and AA+CKD groups when compared to the untreated and AA groups. Total bilirubin showed minimal changes across groups and largely remained within reference ranges, and CHOL was elevated in CKD (175 mg/dL) and AA+CKD female HPP mice (157 mg/dL) (Table S2).

### A‌A supports skeletal health in the late-onset HPP model, but it does not fully rescue bone deficits when CKD is present

Whole limb radiographical images revealed marked skeletal abnormalities in female and male HPP mice, with significant shortening of the femur but not the tibia when compared to non-HPP controls. Female HPP mice showed reduced femur lengths, which were not rescued by AA alone or when AA was associated with CKD. Tibia length remained largely unchanged across groups, except for an increased length in the female non-HPP AA relative to the non-HPP+CKD group. Longer length of the tibia was observed in non-HPP AA compared to the HPP AA-treated group and to the non-HPP CKD cohort (Figure S3). A similar pattern was observed in male mice, where AA did not improve bone length in HPP alone and did not provide benefits in the presence of CKD (Figure S4). These findings indicate that although AA supports overall bone growth in HPP, in the studied regimen, it did not rescue long bone lengths when administered alone or in combination with CKD, and its efficacy in restoring bone length is markedly compromised in the presence of CKD.

**Figure 2:**
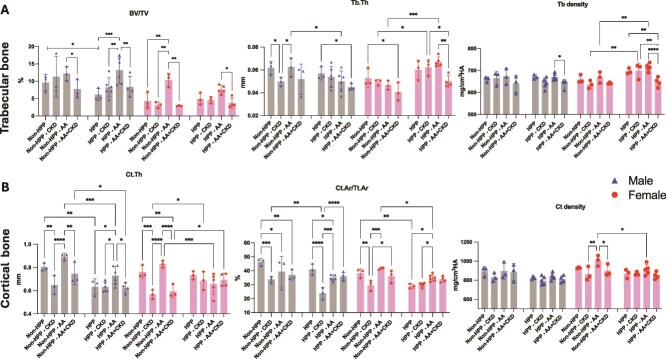
Micro-CT analysis of trabecular and cortical bone parameters in HPP mice with or without CKD and asfotase alfa treatment. Trabecular (A) and cortical (B) bone microarchitecture and mineral density were quantified by micro-CT in femora from male (blue triangles) and female (red circles) mice across experimental groups. Assessed trabecular parameters include bone volume fraction (BV/TV), trabecular thickness (Tb.Th), and trabecular tissue mineral density (Tb.density). Cortical measurements include cortical thickness (Ct.Th), cortical area fraction (Ct.Ar/Tt.Ar), and cortical tissue mineral density (Ct.density). Bars represent mean ± SD. Group comparisons were evaluated using one-way ANOVA followed by Tukey’s post hoc test. ^*^p < .05, ^**^p < .01, ^***^p < .001, ^****^p < .0001. *N* = 3-6.

Micro-CT analysis revealed significant deficits in both trabecular and cortical bone parameters in HPP mice compared to non-HPP controls (Figure 2A and B). Untreated male HPP mice exhibited markedly reduced trabecular bone volume fraction (BV/TV) and compromised cortical thickness (Ct.Th). In contrast, female HPP mice showed a reduced cortical area fraction (Ct.Ar/Tt.Ar) compared with non-HPP controls. Treatment with AA led to significant improvements in BV/TV and Ct.Th, and partially rescues both trabecular and cortical bone parameters. However, when CKD was superimposed on HPP mice undergoing long-term AA treatment (HPP+AA+CKD), the bone phenotype significantly worsened. HPP+AA+CKD mice displayed lower BV/TV, Tb.Th, Tb density, and Ct.Th, than AA alone (Figure 2A and B). Micro-CT reconstructions (2D and 3D) of the femur reinforce the quantitative results in Figure 2, revealing structural bone differences across groups and demonstrating that HPP mice exhibit marked skeletal defects compared to controls, which are further exacerbated by CKD-induced bone deterioration (Figure S5).

### Increased renal inflammation and calcification in AA-treated HPP mice with CKD

CD45 is a well-known marker of CKD severity and progression risk.^21^ Histological evaluation of kidney sections using anti-CD45 immunohistochemistry revealed minimal immune cell infiltration and preserved renal architecture in HPP-untreated and AA-treated mice (Figure S6). In contrast, males presented increased CD45+ cells in the CKD group, and females in the AA+CKD group exhibited increased infiltration of CD45^+^ immune cells when compared to control (Figure S6). These results indicate early signs of fibrosis and progressive tissue remodeling. Notably, the CKD and AA+CKD groups exhibited the most severe inflammatory response and structural disruption in the kidneys.

**Figure 3:**
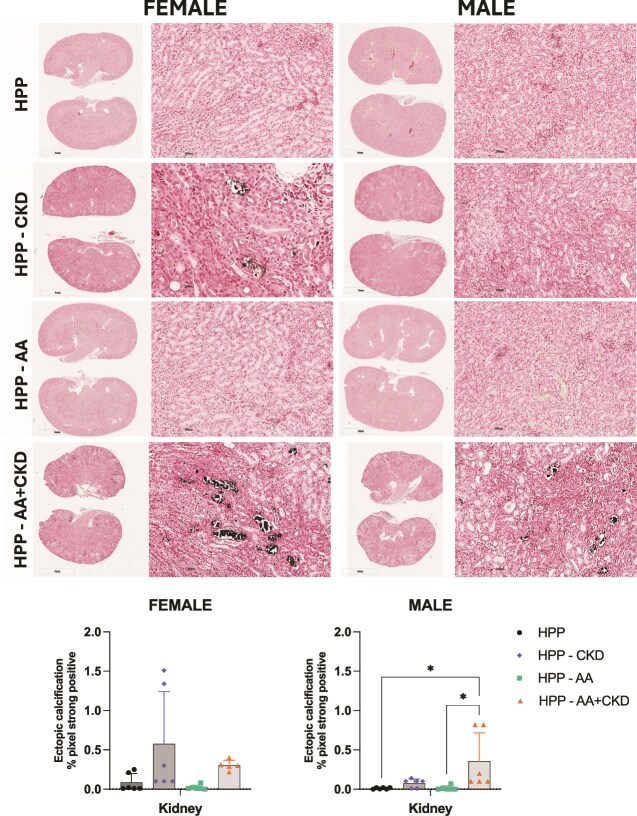
Von Kossa staining of kidney sections from female and male HPP mice. Representative low-magnification (1×) and high-magnification (20×) images of kidney sections from HPP, HPP+CKD, HPP+AA, and HPP+AA+CKD groups. Sections were stained using the Von Kossa method, in which phosphate-containing mineral deposits appear black. Images are shown for both sexes and include whole-kidney overviews (left panels) and higher-magnification cortical/interstitial regions (right panels). *N* = 12. Quantification of ectopic calcification in kidney sections from HPP mice under different treatment conditions was performed using Von Kossa staining. Kidney sections are shown, with quantification of mineral deposition expressed as the percentage of strongly positive pixels per tissue area across the HPP, HPP+CKD, HPP+AA, and HPP+AA+CKD groups. ^*^p < .05, ^**^p > .01, ^***^p < .001, ^****^p < .0001.

Von Kossa staining of kidney sections revealed calcium deposits indicating ectopic calcification mainly in the AA+CKD group, in male HPP mice (Figure 3). A few spots of mineral deposition were observed in most kidneys from CKD-only and AA+CKD female mice, but no significant differences were observed. Renal calcification was not observed in the AA or untreated groups in either female and male mice (Figure 3).

**Figure 4:**
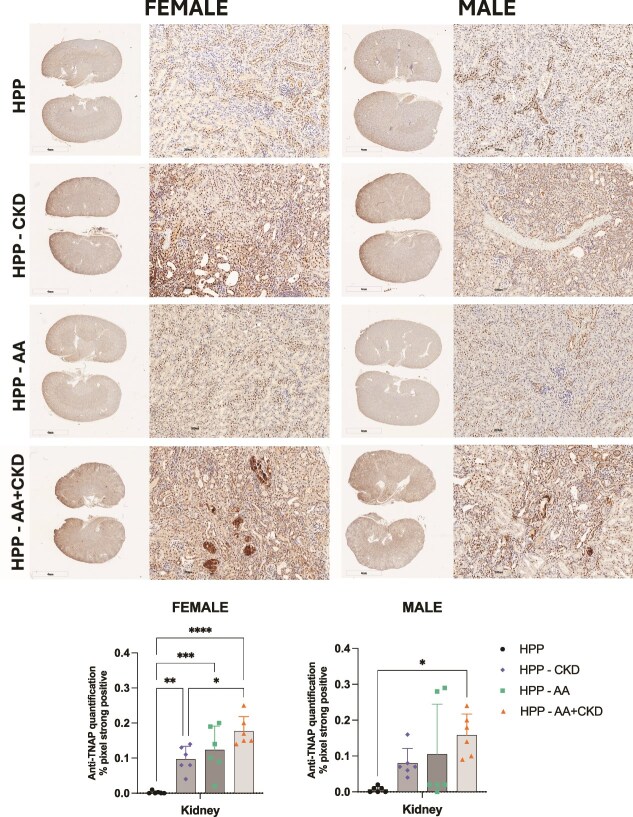
Immunohistochemical localization of tissue-nonspecific alkaline phosphatase (TNAP) in the kidneys of female and male mice across experimental groups. Representative kidney sections from HPP, HPP+CKD, HPP+AA, and HPP+AA+CKD groups stained with anti-TNAP antibody. Tissue-nonspecific alkaline phosphatase expression is visualized as a brown chromogenic signal. Images include whole-kidney views (left panels) and higher-magnification regions (right panels) for each sex. *N* = 12. Quantification of TNAP immunostaining in kidney sections from HPP mice under different treatment conditions was measured by quantifying TNAP-positive areas, expressed as the percentage of strongly positive pixels per total tissue area, in the HPP, HPP+CKD, HPP+AA, and HPP+AA+CKD groups. ^*^p < .05, ^**^p > .01, ^***^p < .001, ^****^p < .0001.

Immunohistochemical staining for TNAP showed minimal localization in the kidneys of untreated female and male HPP mice. In contrast, CKD, AA, and AA+CKD groups showed widespread strong TNAP staining in female HPP kidneys (*p* < .05). Although TNAP staining was observed in male HPP CKD, AA, and AA+CKD-treated groups, only AA+CKD HPP mice showed a significant difference compared with untreated mice (*p* < .05) (Figure 4). It is important to note that this antibody showed some nonspecific nuclear staining. However, it was effective for identifying areas associated with mineral deposition.

### Cardiovascular calcification is enhanced in AA-treated HPP mice with comorbid CKD

**Figure 5:**
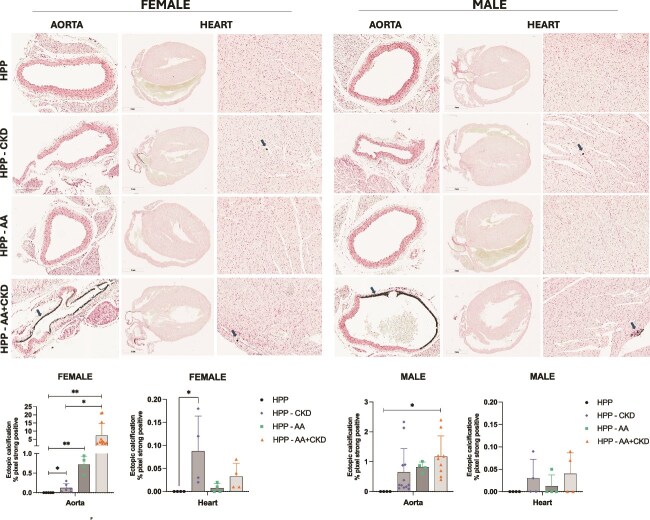
Von Kossa staining of aorta and heart sections from female and male HPP mice. Representative aortic and cardiac sections from the HPP, HPP+CKD, HPP+AA, and HPP+AA+CKD groups were stained with Von Kossa to visualize phosphate-containing minerals (black staining). Aortic images are shown at 20× magnification (*N* = 6-13), and heart images are shown at both 1× and 20× magnification (*N* = 8). Quantification of ectopic calcification in aorta and heart sections from HPP mice under different treatment conditions was performed by Von Kossa staining. Aorta (left panel) and heart (right panel) sections are shown with quantification of mineral deposition, expressed as the percentage of strongly positive pixels per total tissue area across HPP, HPP+CKD, HPP+AA, and HPP+AA+CKD groups. ^*^p < .05, ^**^p < .01.

Von Kossa staining revealed no evidence of vascular or cardiac calcification in the untreated HPP mice (Figure 5). In contrast, AA+CKD mice displayed massive, distinct black deposits in the aortic wall, indicative of vascular calcification, observed in both sexes. CKD-only and AA-only female groups presented ectopic calcification related to untreated HPP mice (*p* < .05), although at lower levels than in the CKD-AA group and in males no significant difference was found comparing CKD or AA only with untreated HPP mice. Additionally, sporadic areas of myocardial calcification were detected in the hearts of CKD mice, with a significant difference only observed in females (Figure 5).

**Figure 6:**
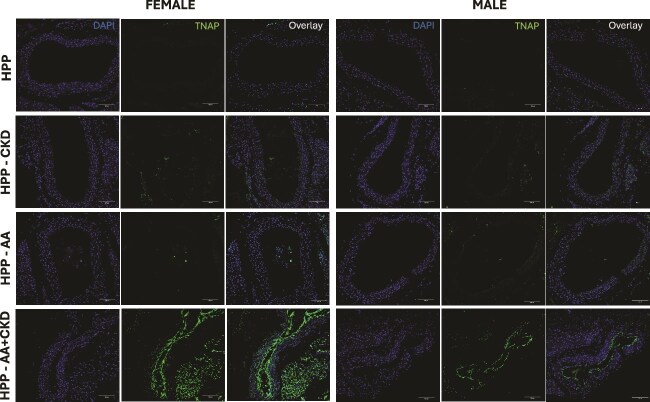
Immunofluorescence staining of TNAP in aortic sections from female and male HPP mice. Representative aortic sections from HPP, HPP+CKD, HPP+AA, and HPP+AA+CKD groups stained with anti-TNAP antibody (green) and counterstained with DAPI for nuclei (blue). Panels show TNAP, DAPI, and merged overlay images for each sex. Images were acquired at 20× magnification. Scale bar = 100 μm. *N* = 3.

Immunofluorescence analysis of aortic sections revealed minimal TNAP expression in HPP-untreated mice of both sexes (Figure 6). Tissue-nonspecific alkaline phosphatase fluorescence levels remained low in mice subjected to CKD or AA alone, with only modest increases in the aortic wall or adjacent areas. In contrast, the combination of AA and CKD markedly enhanced TNAP expression in both female and male mice. In females, TNAP staining was robust and widespread and broadly distributed throughout the aortic media and surrounding structures, while in males, TNAP expression appeared more focal and closer to the luminal side of the vessel, suggesting predominant localization in the intima (Figure 6).

### Differential renal gene expression in fibrotic and inflammatory pathways

**Figure 7:**
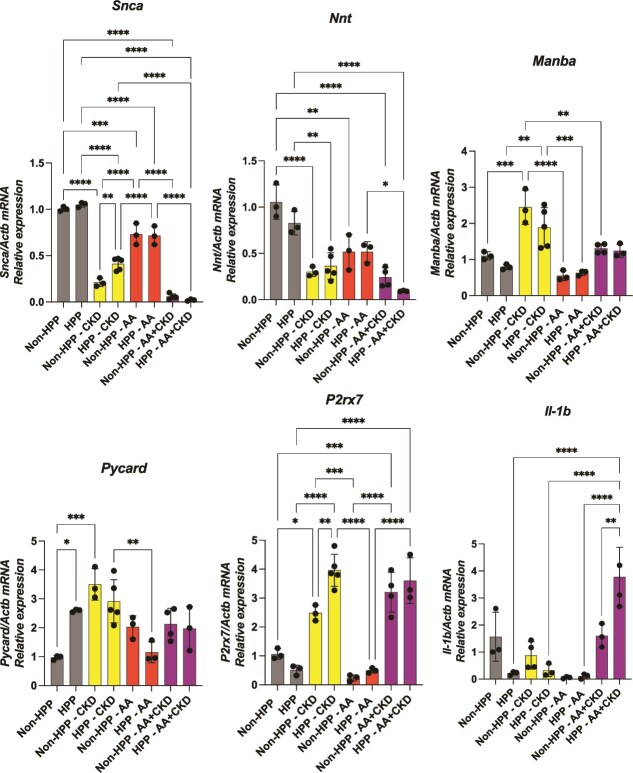
Relative mRNA expression of oxidative stress, lysosomal function, and inflammasome-related genes in kidney tissue from non-HPP and HPP mice. Quantitative PCR was performed to measure the expression of *Snca*, *Nnt*, *Manba*, *Pycards*, *P2rx7*, and *Il-1b* in kidney samples from male non-HPP controls and HPP mice under CKD, AA, and AA+CKD conditions. Gene expression values were normalized to *Actb* and are displayed as mean ± SD. Statistical comparisons were conducted by one-way ANOVA followed by Tukey’s post hoc test (^*^p < .05, ^**^p < .01, ^***^p < .001, ^****^p < .0001). *N* = 3.

Renal gene expression analysis by RT-qPCR in males revealed differential expression of genes associated with fibrosis, mitochondrial antioxidant defense, inflammation, and lysosomal and immune function across experimental groups (Figure 7). Synuclein alpha (Snca) and nicotinamide nucleotide transhydrogenase (Nnt) expression were significantly downregulated in CKD and AA+CKD non-HPP and HPP mice compared to untreated non-HPP and HPP mice, suggesting an association with fibrotic changes in dilated renal tubules^22^ and with mitochondrial anti-oxidant defense.^23^ Mannosidase Beta (Manba) expression, which is linked to lysosomal function,^24^ was markedly increased in non-HPP-CKD and HPP-CKD mice when compared to untreated non-HPP and HPP mice, but AA attenuated this induction. Regarding inflammasome-related genes, PYD and CARD Domain Containing (Pycard), encoding the ASC adaptor protein,^25^ was significantly upregulated in the non-HPP CKD group when compared to untreated non-HPP. In addition, *Pycard* was upregulated in HPP control mice relative to non-HPP mice. Purinergic receptor P2X 7 (P2rx7), an ATP-gated ion channel involved in cytokine release,^26^ was significantly upregulated in CKD conditions and strongly suppressed by AA alone HPP and non-HPP. Interleukin 1 beta (Il-1b), a pro-inflammatory cytokine downstream of inflammasome activation, showed marked upregulation in HPP AA+CKD mice. Untreated, CKD, and AA HPP mice showed strong suppression of IL-1b compared to HPP AA+CKD, demonstrating that combined AA+CKD promoted a release of proinflammatory cytokine (Figure 7). Overall, the gene expression analysis revealed a pattern characterized by increased expression of inflammatory and lysosomal-related genes and decreased expression of genes associated with mitochondrial function and antioxidant defense associated with the CKD and AA+CKD cohorts. These changes were more pronounced in AA+CKD groups, particularly in HPP mice, indicating a consistent treatment-associated gene expression profile across groups.

## Discussion

Asfotase alfa, a recombinant mineral-targeted form of TNAP, has demonstrated significant efficacy in improving survival, growth, and bone mineralization for pediatric-onset HPP, particularly in patients with the severe perinatal or infantile forms.^3^ However, its therapeutic application in adults with late-onset HPP remains controversial due to clinical heterogeneity, a lack of long-term efficacy data, and the high cost of the treatment.^27^ Our study begins to address the potential negative associations between long-term AA therapy in HPP patients who may develop comorbid conditions associated with ectopic calcification as these patients age, undergoing lifelong treatments.

We purposely chose a dosing regimen aimed at maintaining a state of ameliorated but not fully corrected skeletal disease so that the effects of introducing CKD would become apparent. This approach is analogous to examining enzyme inhibition when working at substrate concentrations close to Km (corresponding to one-half Vmax), where catalytic activity readily changes with perturbations, rather than under Vmax conditions, where enzyme activity has reached full capacity.^28^ We opted for 3 times per week injections of AA since in pilot experiments we determined that the ALP levels achieved were in the range obtained earlier when using 4 × 10^8^ viral particles per body of AAV8-TNAP-D_10_ construct,^16^ a dose that ameliorated but not fully corrected the skeletal defects in the *Alpl*^*Prx1/*−^ mouse model of late-onset HPP. This AA treatment regime in the HPP mice led to rather large increases in serum ALP levels in males but very modest increases in female mice. The reasons for this sexually dimorphic response are not clear, but the data would suggest that the administered AA is cleared much more slowly by the Ashwell Morell receptor^29^ in the liver in male than in female mice. Interestingly, CKD induction resulted in further elevations of ALP, particularly in female mice, and this effect was due to the upregulation of endogenous *Alpl* gene expression in the conditional *Alpl^Prx1/−^* mouse model, which occurred despite the mice harboring one null allele. This reflects an intrinsic limitation of this model, because it is only the mesenchymal stem cells lineage that ablated the function of the remaining functional allele via breeding the *Alpl^flox/−^* mice to the *Prx1-Cre* deleter mouse strain. In the future, we may need to assess if a similar upregulation of kidney-derived TNAP expression might occur in mouse models that are compound heterozygous for hypomorphic *Alpl* alleles, as the elevations in serum ALP activity, even if they occur, would be expected to be significantly less pronounced.

Inorganic pyrophosphate levels were lower in the HPP AA-treated groups of females and males, reflecting enhanced hydrolysis by TNAP. As TNAP cleaves PP_i_ into inorganic phosphate (P_i_), this aligns with the mechanism of action. Elevated serum phosphorus in CKD and AA+CKD groups, attributable to the phosphate-enriched diet, reflects impaired renal excretion of P_i_, a hallmark of CKD-MBD.^30^^,^^31^ The accumulation of P_i_, in conjunction with diminished PP_i_, clearly creates a pro-calcific environment, particularly in soft tissues.

Micro-CT analyses confirmed that AA partially restored bone length and microarchitecture in HPP mice. However, these benefits were not observed in the presence of CKD, and AA+CKD mice even exhibited significantly shorter long bones and more severe deterioration of trabecular and cortical bone parameters than treated HPP mice. CKD is known to disrupt mineral metabolism, increase pro-inflammatory cytokines, and elevate PTH levels,^13^ all of which contribute to bone resorption and impaired mineralization.^8^

Histological analysis of the kidneys revealed minimal inflammation in untreated and AA-treated HPP mice. In contrast, CKD and AA+CKD groups showed marked immune cell infiltration, tubular dilation, and fibrosis, with the most severe pathology observed in AA+CKD female mice and in CKD male mice. Despite our data showing ectopic calcification on CKD and AA+CKD HPP female mice, no significant difference was found compared to untreated HPP mice. These findings suggest that TNAP, either as AA accumulation in the kidneys or *Alpl* upregulation, or a combination of both, in the context of renal dysfunction, may aggravate local inflammation and fibrotic remodeling. Tissue-nonspecific alkaline phosphatase is known to be upregulated in calcifying vascular and renal tissues and can promote mineral deposition when PP_i_ levels are low.^32^ Consequently, the administration of AA and the upregulation of endogenous *Alpl* may have driven nephrocalcinosis and structural kidney injury during this treatment. Von Kossa staining confirmed calcium deposits in the kidneys of AA+CKD HPP mice, supporting the hypothesis of ectopic calcification driven by altered phosphate metabolism and the excess TNAP activity. Immunohistochemical staining further demonstrated increased TNAP expression in renal tissues, with the highest levels observed in the AA+CKD group. Prior work has shown that TNAP promotes hydroxyapatite deposition in vascular smooth muscle cells and renal vasculature under pro-calcification conditions,^8^^,^^11^ reinforcing our findings. Similar calcified lesions were detected in the vascular tissue of female CKD, AA, and AA+CKD mice, and in male HPP mice, with the AA+CKD group, and in cardiac tissue, ectopic calcification was found, with a significant difference only in CKD female HPP mice. These observations point to the need to carefully evaluate disease progression and treatment efficacy in HPP patients when comorbid ectopic calcification may be present.

Recent clinical observations further underscore the clinical importance of our findings. A lifetime follow-up of a woman with pediatric-onset HPP complicated by advanced CKD showed that long-term management is particularly challenging when skeletal disease coexists with renal dysfunction.^33^ This patient required repeated orthopedic interventions, developed end-stage renal disease, and ultimately benefited from kidney transplantation, which appeared to improve bone outcomes. Importantly, temporary treatment with the PTH analogue teriparatide enhanced bone mineralization and alleviated pain, but the effects were not durable, emphasizing the limited options for adult HPP patients with CKD.^33^ In line with our experimental data, these clinical findings suggest that renal impairment exacerbates skeletal fragility in HPP and may blunt the efficacy of bone-targeted therapies, such as enzyme replacement, thereby highlighting the need to evaluate treatment strategies within the context of comorbid CKD.

Importantly, sex-specific differences were observed throughout the study. Male mice consistently showed higher ALP levels and distinct hepatic and skeletal responses to AA and CKD. Sex hormones are known to have regulatory effects on bone turnover, renal phosphate handling, and inflammatory responses, which may account for these differences.^34^ Future work should explore sex hormones and their interaction with TNAP biology to understand differential susceptibility to AA response. These findings emphasize that while AA improves bone outcomes in HPP subjects, its therapeutic effects may be dampened when experiencing comorbid CKD. Clinically, this suggests that adult HPP patients with even mild renal impairment may manifest a reduced benefit from the standard therapy, and monitoring renal function, mineral balance, and inflammation might be indicated.

Several limitations should be acknowledged. Small sample sizes in certain datasets represent a limitation that may influence the robustness and generalizability of these results. This study was conducted in a genetically modified mouse model (*Alpl^Prx1/−^*), which may not fully recapitulate the complexity of late-onset HPP in humans, especially concerning the diversity of *ALPL* mutations and clinical variability.^35^ The CKD model utilized a high-phosphate adenine diet, which induces tubulointerstitial nephropathy but may not reflect all aspects of human CKD pathophysiology. The dose and frequency of AA administration may not correspond precisely to therapeutic regimens that would be adopted in the clinical setting. Finally, the observational period was restricted to four months of treatment, and longer-term outcomes, particularly regarding calcification progression or reversibility, remain unknown. Future studies, including time-course analyses, pharmacokinetics, and broader histopathological evaluations, will be needed to validate these findings and translate them to clinical settings.

In summary, this study provides mechanistic insight into the dampened effect of AA therapy in the setting of renal dysfunction as comorbidity in an HPP preclinical model and identifies systemic inflammatory and calcification signatures suggesting potential novel biomarkers for monitoring disease progression and therapeutic response in HPP with comorbid CKD.

## Supplementary Material

Supplemental_Material-041326_ziag074

## Data Availability

The data that support the findings of this study are available in the supplementary material of this article. Additional data related to this study might be available upon request to the authors.
