# Supplementary material for: Effects of comorbid chronic kidney disease on late-onset hypophosphatasia mice under treatment with asfotase alfa
Source: JBMR Plus. 2026 Apr 17;10(6):ziag074. doi: 10.1093/jbmrpl/ziag074 (PMC13171041; doi:10.1093/jbmrpl/ziag074)
Supplement: Supplemental_Material-041326_ziag074 [file supplemental_material-041326_ziag074.pdf]

Supplemental Material

Suppl. Table 1: Primer sequence.

**Primers (mouse)**		**Forward primer (F)**			**Reverse primer (R)**	
	**5'**		**3'**	**5'**		**3'**
Alpl		ATTCCCACTATGTCTGGA			CTCAAAGAGACCTAAGAG	
Il-1b		GGATGATGATGATAACCTGC			CATGGAGAATATCACTTGTTGG	
Manba		AAGCAGCCAAGGTTTATTTC			ATCCAATTTGAGCCTTTCAG	
Nnt		TTCTGGTGACTCAGAGAATG			CCAGGTACATGATTTCTTCG	
P2rx7		ATCGAGATCTACTGGGATTG			GTACTTGGCATATCTGAAGTTG	
Pycard		AATTTAACATGGGTTTCCCC			AGTTTGTCTTCAAGCTTCTG	
Snca		TTATGAAATGCCTTCAGAGG			TGGAAGACTTTGAAACACAC	

Suppl. Table 2: Whole blood biochemical parameters measured using the Abaxis VetScan rotor.

Parameters	**Non-HPP**							
	**Non-HPP**	**CKD**	**AA**	**AA+CKD**	**Non-HPP**	**CKD**	**AA**	**AA+CKD**
	**Female**				**Male**			
ALT (U/L)	248±43	196±59	197±48	980±117	187±44	59±16	35±12	937±251
GGT (U/L)	<5	<5	<5	69±31	<5	6±1	<5	0
BA (umol/L)	2±3	13.7±1	<1	19±1	<1	3.7±2	1.3±2	35±26
TBIL (mg/dL)	0.3±0	0.3±0	0.3±0	0.2±0	0.3±0	0.3±0	0.37±0	0.2±0
BUN (mg/dL)	24±4	52±13	22±3	99±8	19±3	64±11	20±4	151±25
CHOL (mg/dL)	94±12	78±25	97±11	141±25	126±12	135±20	108±12	136±47

Parameters	**HPP**							
	**HPP**	**CKD**	**AA**	**AA+CKD**	**HPP**	**CKD**	**AA**	**AA+CKD**
	**Female**				**Male**			
ALT (U/L)	145±19	209±36	105±32	93.5±23	118±54	401±74	48±17	255±44
GGT (U/L)	<5	5±1	<5	<5	<5	5.5±1	<5	<5
BA (umol/L)	<1	11±1	<1	12.3±2	<1	8.3±5	<1	3.7±2
TBIL (mg/dL)	0.3±0	0.3±0	0.3±0	0.2±0	0.3±0	0.2±0	0.3±0	0.2±0
BUN (mg/dL)	25.3±5	148±29	23±2	107.3±9	24.7±2	86±17	20.3±3	100±24
CHOL (mg/dL)	98±4	175±9	97±6	157±6	96.3±28	151±18	136±14	127±23

Reference range: ALT (U/L) 17-77; GGT (U/L) 6-9; BA (umol/L) ---; TBIL (mg/dL) 0.2 -0.5; BUN (mg/dL) 8-33; CHOL (mg/dL) 81-135. Data is shown as mean ±SD. N=3. Values were interpreted relative to the reference ranges provided for the assay rather than for statistical comparison between groups.

Supplementary Figures

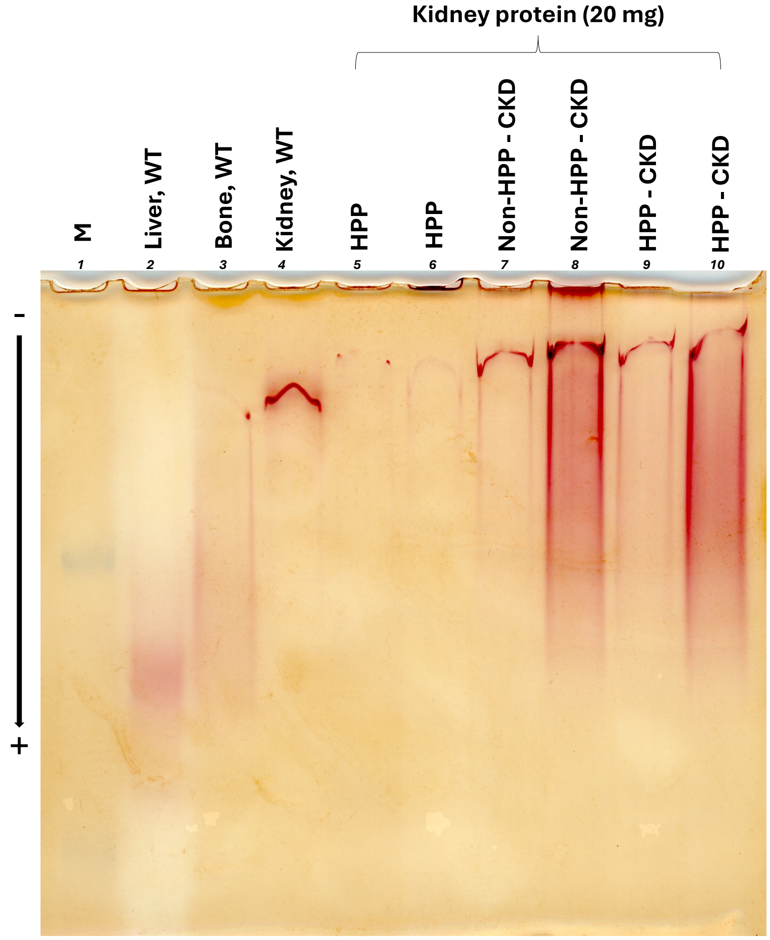

Supplementary Figure 1. Native gel electrophoresis of alkaline phosphatase (ALP) activity in tissue extracts from WT, HPP, and Non-HPP CKD mice. Tissue samples were homogenized, extracted using the butanol extraction method, and protein concentration was determined by the BCA assay. To compare AP migration patterns, extracts from liver, bone, and kidney of WT mice (lanes 2-4; adjusted with AP activity and variable protein concentrations) were analyzed alongside kidney extracts from HPP mice (lanes 5, 6, N=2), non-HPP mice with CKD (lanes 7, 8, N=2), and HPP mice with CKD (lanes 9, 10, N=2). Twenty micrograms of total protein were loaded per lane, and ALP activity was visualized using the Azo Dye coupling method. M: molecular weight marker; the blue band corresponds to a 250 kDa protein. ALP activity in CKD samples showed a smeared

migration pattern originating from the slow-migrating region, indicative of altered ALP isoform distribution.

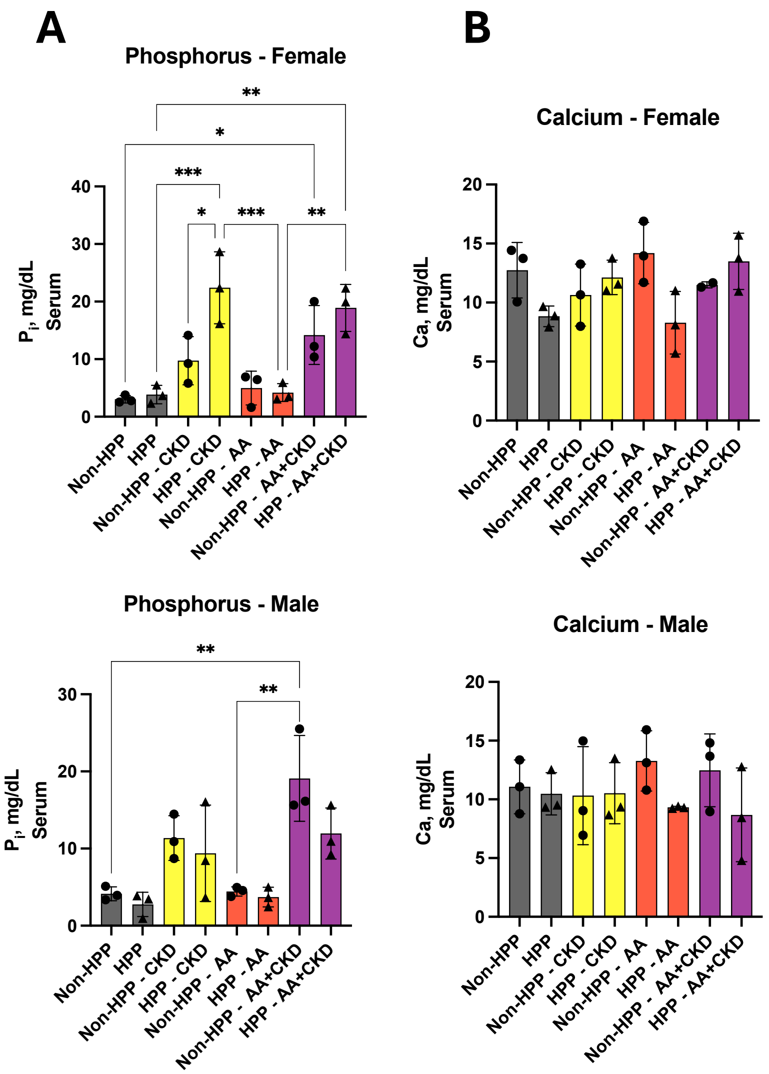

Supplementary Figure 2. Biochemical response to asfotase alfa in a late-onset HPP mouse model with superimposed CKD. (A) Serum phosphorus levels in female and male mice across all experimental groups. (B) Serum calcium levels in female and male mice. Data represent individual values and mean ± SD. N = 3 per group.

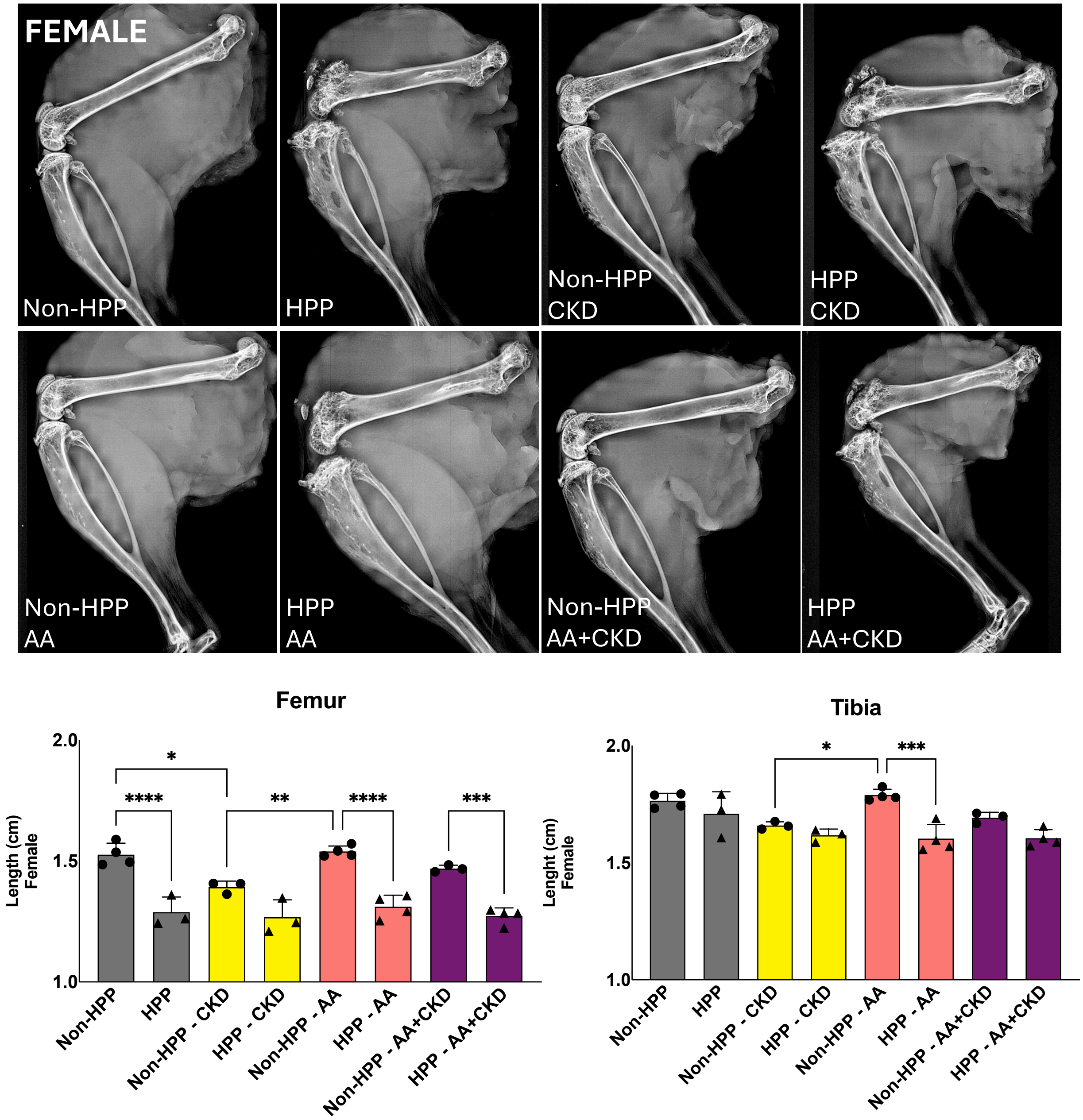

Supplementary Figure 3. Radiographic analysis of femur and tibia length in female mice. Representative radiographs of hindlimbs from all experimental groups, including Non-HPP, HPP, Non-HPP+CKD, HPP+CKD, Non-HPP+AA, HPP+AA, Non-HPP+AA+CKD, and HPP+AA+CKD. Bar graphs show quantification of femur and tibia lengths (mean ± SD, N = 3-4). Statistical comparisons were performed using one-way ANOVA followed by Tukey's post hoc test. *$p<0.05$, **$p<0.01$, ***$p<0.001$, ****$p<0.0001$.

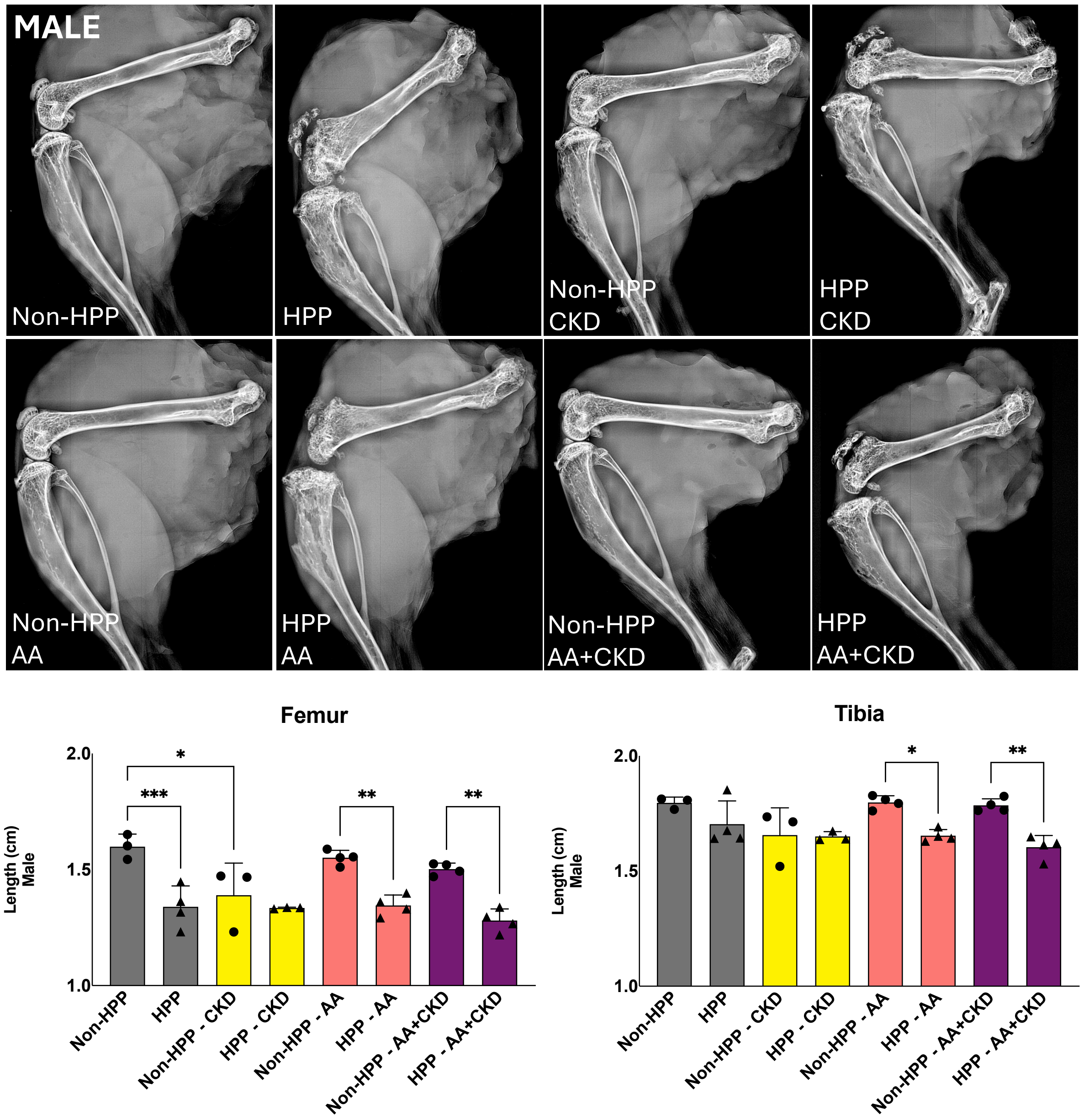

Supplementary Figure 4. Radiographic analysis of femur and tibia length in male mice. Representative radiographs of hindlimbs from all experimental groups, including Non-HPP, HPP, Non-HPP+CKD, HPP+CKD, Non-HPP+AA, HPP+AA, Non-HPP+AA+CKD, and HPP+AA+CKD. Bar graphs show quantification of femur and tibia lengths (mean ± SD, N = 3-4). Statistical comparisons were performed using one-way ANOVA followed by Tukey's post hoc test. *$p<0.05$, ***$p<0.001$.

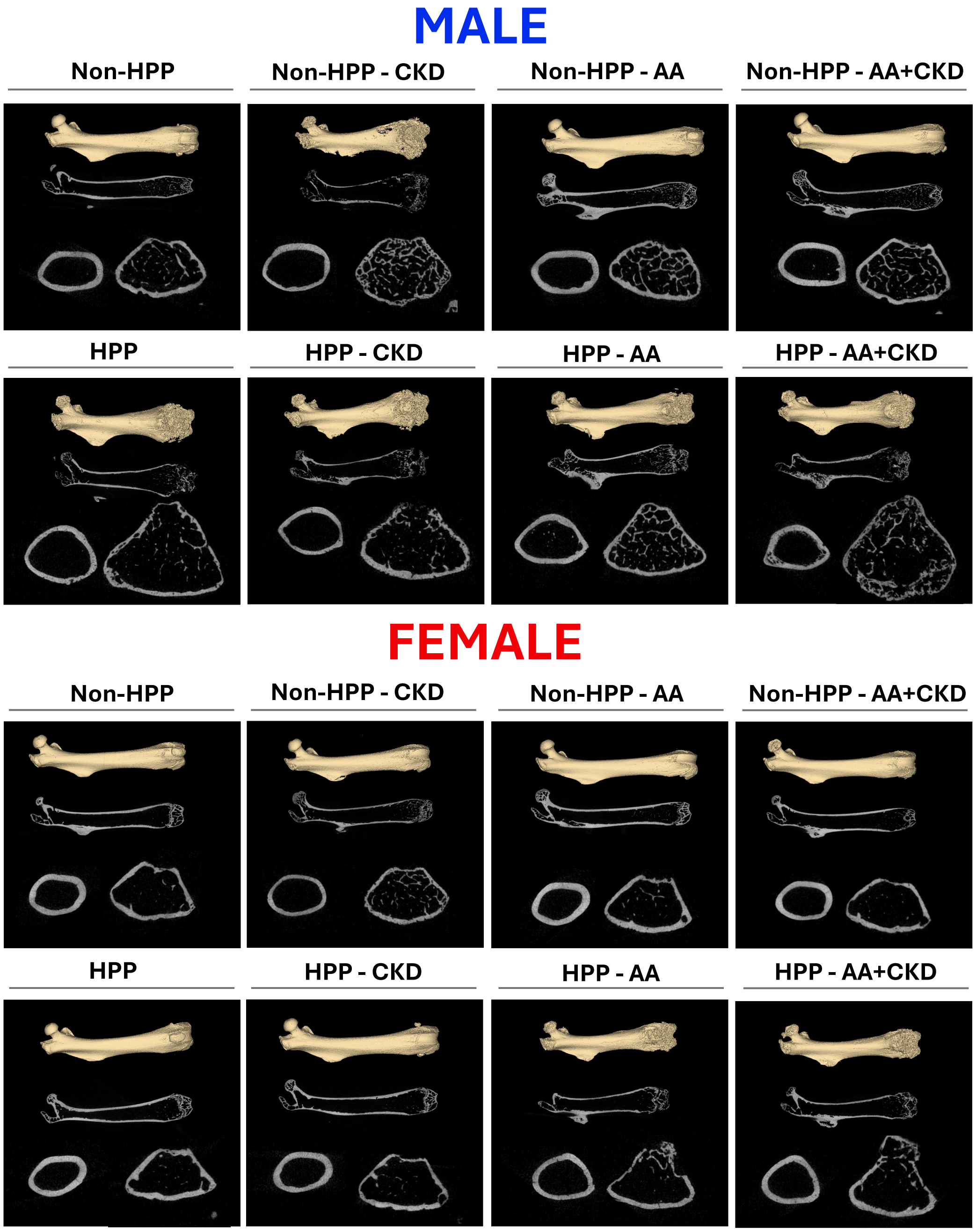

Supplementary Figure 5. Representative 2D and 3D micro-CT images of femora from male and female mice. Micro-CT reconstructions show transverse and longitudinal views of femora from all experimental groups in males (top panel) and females (bottom panel). Images include Non-HPP, Non-HPP+CKD, Non-HPP+AA, Non-HPP+AA+CKD, HPP, HPP+CKD, HPP+AA,

and HPP+AA+CKD groups. Reconstructions illustrate cortical and trabecular bone morphology for qualitative comparison.

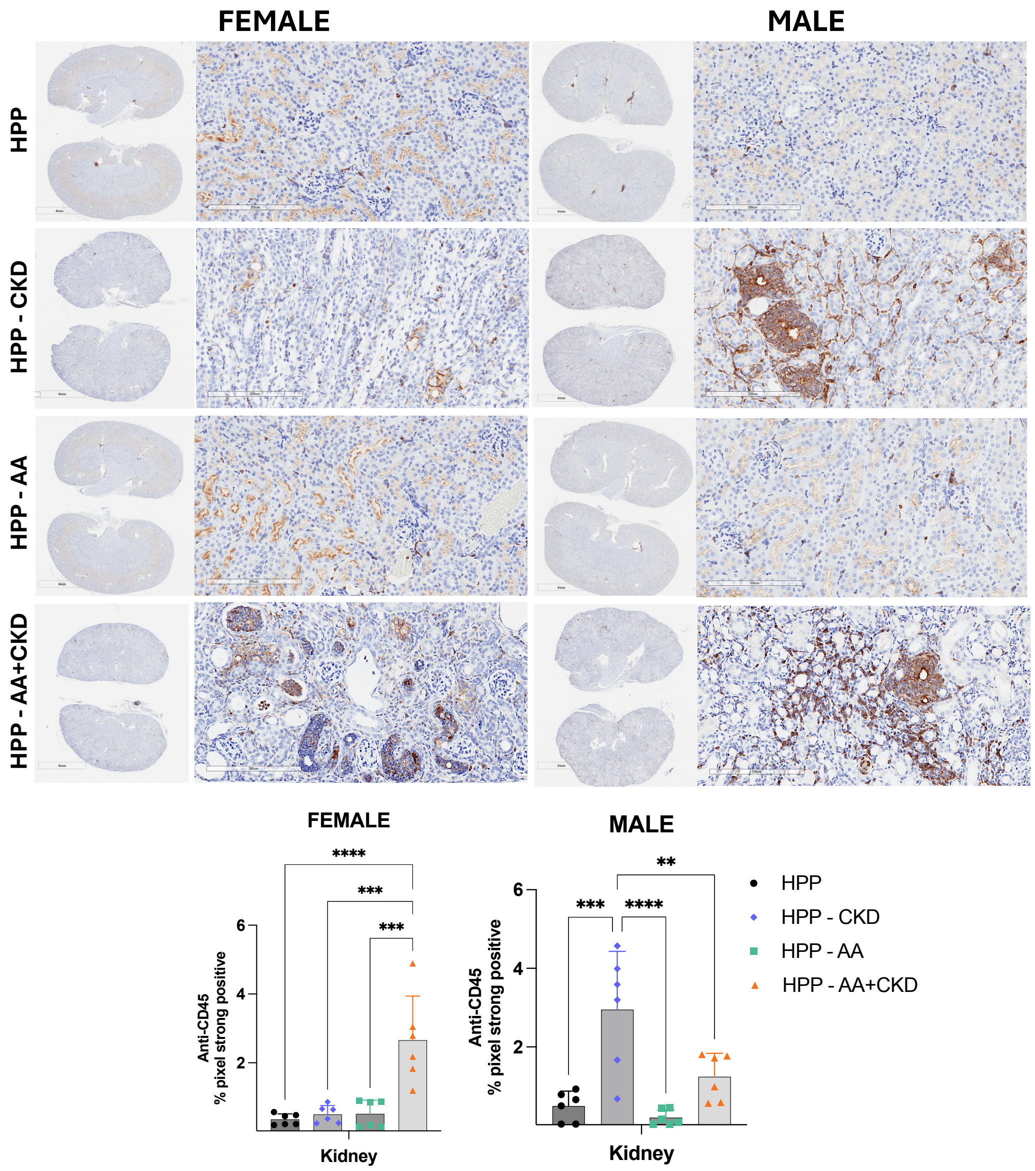

Supplementary Figure 6. CD45 immunohistochemistry in kidney sections from female and male HPP mice. Representative low magnification (1x) whole kidney sections and high magnification (20x) images of renal cortex from female and male mice across the HPP, HPP+CKD, HPP+AA, and HPP+AA+CKD groups. Quantification of CD45-positive staining is shown as the percentage of strongly positive pixels per total tissue area and represents pooled data from female and male mice. **p<0.01, ***p<0.001, ****p<0.0001.
